# A diffusion model analysis of age and individual differences in the retro-cue benefit

**DOI:** 10.1038/s41598-023-44080-z

**Published:** 2023-10-13

**Authors:** Alessandra S. Souza, Gidon T. Frischkorn

**Affiliations:** 1 Center for Psychology, Faculty of Psychology and Education Sciences, University of Porto, Rua Alfredo Allen S/N, 4200-135 Porto, Portugal; 2 University of Zurich, Zürich, Switzerland

**Keywords:** Human behaviour, Cognitive ageing

## Abstract

The limited capacity of working memory (WM) constrains how well we can think and act. WM capacity is reduced in old age, with one explanation for this decline being a deficit in using attention to control WM contents. The retro-cue paradigm has been used to examine the ability to focus attention in WM. So far, there are conflicting findings regarding an aging deficit in the retro-cue effect. The present study evaluated age-related changes and individual differences in the retro-cue effect through a well-established computational model that combines speed and accuracy to extract underlying psychological parameters. We applied the drift–diffusion model to the data from a large sample of younger and older adults (total N = 346) that completed four retro-cue tasks. Retro-cues increased the quality of the evidence entering the decision process, reduced the time taken for memory retrieval, and changed response conservativeness for younger and older adults. An age-related decline was observed only in the retro-cue boost for evidence quality, and this was the only parameter capturing individual differences in focusing efficiency. Our results suggest that people differ in how well they can strengthen and protect a focused representation to boost evidence-quality accumulation, and this ability declines with aging.

## Introduction

**Figure 1:**
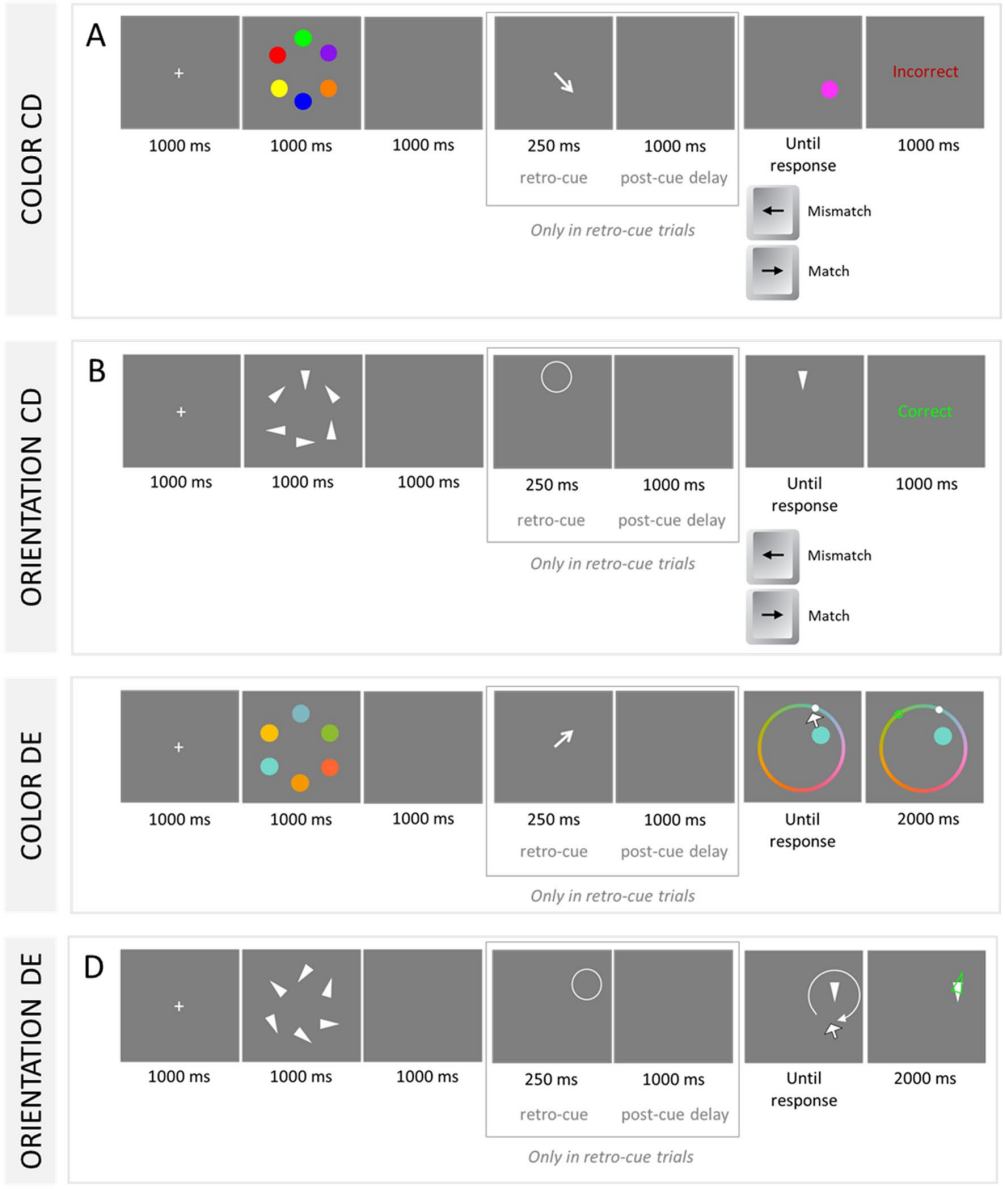
Illustration of key features of retro-cue tasks used in the present study. These tasks varied the nature of the memory test (change detection, CD vs. delayed estimation, DE), the memoranda (orientation vs. color), and the type of retro-cue (central arrow vs. peripheral circle).

Working memory provides a limited workspace to represent the information guiding our thoughts and actions. Its capacity decreases during healthy aging, but the causes of this decline are still unclear. One candidate explanation relates to deficits in the use of attention to control working memory contents. Attention can be used to flexibly prioritize and update working memory contents to reflect only the most relevant ones for adaptive action. This process has been studied using the *retro-cue* paradigm^1,2^. In this paradigm, a cue is presented during the retention interval indicating which working memory content will be relevant for the memory test. Take, for example, the task illustrated in Fig. 1A. Participants are asked to memorize the colors of a set of disks. In the standard no-cue condition, the retention interval is followed directly by the test. In retro-cue trials, in contrast, the retention interval is followed by the presentation of a cue highlighting one memory location. The cue draws attention to one working memory content, indicating it as the relevant representation for the memory test. The validity of the retro-cue in predicting the tested item can be varied, but it is usually set at 100% [for a review see 3]. Typically, responses to the memory test are faster and more accurate in valid retro-cue trials than in no-cue trials, yielding the so-called *retro-cue benefit*. The retro-cue benefit has been observed in several paradigms: for example, change detection (see Fig. 1A,B)^1^, change-localization^4^, as well as delayed estimation (see Fig. 1C,D)^5,6^.

### Does aging hinder focusing efficiency in working memory?

The retro-cue effect has been considered one indicator of the efficiency of focusing attention within working memory. Studies have shown that the ability to use the retro-cue increases in childhood^7–9^, yet it is still unclear if it decreases during healthy aging. Some initial studies observed age deficits in the ability to use retro-cues^10,11^, whereas subsequent studies found preserved ability^12–17^. Hence the evidence for an aging deficit in the control of attention in working memory is still mixed. So far, studies have only included relatively small samples (N ≈ 30), and they have only assessed older adults in single tasks. Additionally, tasks across studies varied with regards to their emphasis on response speed or accuracy, which may produce differences on how younger and older adults approach the task. For example, studies finding evidence for an aging deficit in the retro-cue effect used change detection tasks with a response deadline^10,11^, whereas most studies not finding a deficit employed reproduction tasks with an unlimited response window^15,16^. To get a better understanding on how aging affects the ability to focus attention in working memory, it is therefore necessary to include multiple tasks and to combine information from speed and accuracy measures to unravel how people use memory representations to reach a decision. Closing this gap was the main goal of the present study. To achieve this aim, we modeled the data of several retro-cue tasks performed by a large sample of younger and older adults. Next, we explain the rationale of our modeling framework.

### Modeling of the retro-cue effect

One way to gain better insight regarding the operation of focused attention is by examining how it affects parameters of well-established cognitive models. Model parameters have a theory-driven and empirically validated psychological meaning^18^, therefore parameter changes induced by a manipulation can be directly tied to a theoretical interpretation. A well-established class of models in cognitive psychology is of evidence accumulation models. In the present paper, we focus on the diffusion decision model^19–21^ and its recent extension to circular tasks^22–24^.

**Figure 2:**
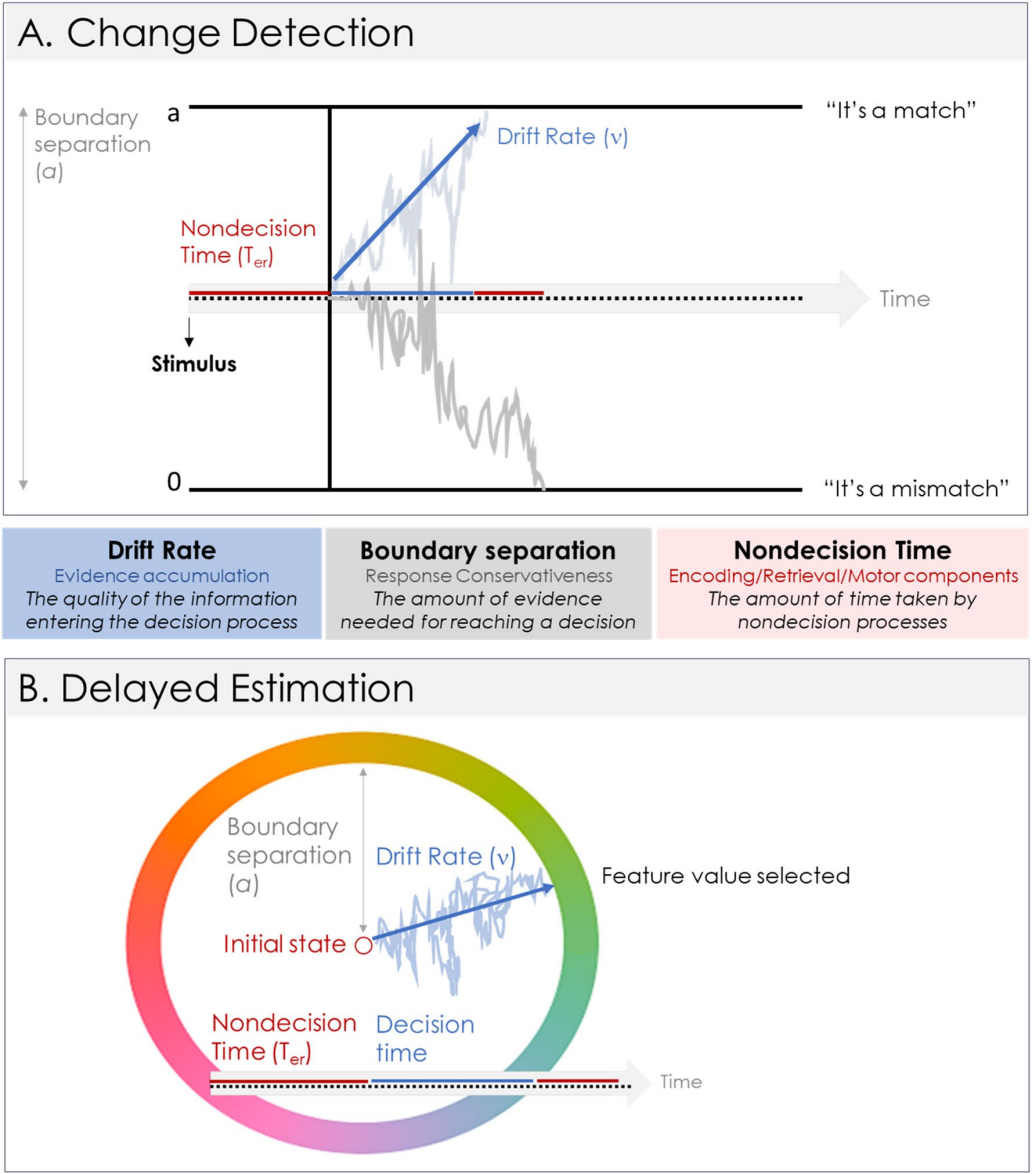
Diffusion model account of the responses in the change detection paradigm (**A**) and the delayed estimation paradigm (**B**). Both model versions have three main parameters: drift rate (ν), boundary separation (a), and nondecision time (T_er_).

These models assume that a decision is made through the gradual and continuous accumulation of evidence towards one out of several response options. This process is illustrated in Fig. 2A in a change-detection paradigm: participants must judge whether a memory probe matches or mismatches a memory item. Its most simplified version—known as the EZ-Diffusion model, which is the model used here^25,26^—describes this process with three parameters. The *drift rate* is the speed of the noisy accumulation of evidence towards a response boundary (i.e., a “match” or “mismatch”), thereby reflecting the quality of the information entering the decision process. The *boundary separation* reflects the evidential distance between the two response options, thereby quantifying how conservatively the participant approaches the task. A larger boundary separation implies that more information needs to be accumulated before a decision is made. Finally, the *nondecision time* parameter captures the time taken by the nondecision processes occurring before (e.g., encoding, memory retrieval) and after the decision is made (e.g., response execution).

This diffusion process has recently been extended to paradigms requiring the precise reconstruction of a feature value in a circular space^23^. The original model is based on the same premises as the two-choice diffusion model described above, and its main parameters are identical as illustrated in Fig. 2B. Recently, a EZ-Continuous Diffusion model^27^ has been proposed that—akin to the EZ-Diffusion model for two-choices—can be applied to summary statistics of the data, requiring only the application of mathematical formulas with no fitting routine. Another advantage of using the EZ versions of these models is that they can be applied to tasks with a relatively low number of trials per condition, and they perform well in recovering individual differences^28^ and simple experimental effects^29^.

To the best of our knowledge, only two previous studies examined the retro-cue effect through the lens of the two-choice diffusion decision model. Shepherdson et al.^30^ modeled the data of four change-detection tasks with visual (colored squares) and verbal (letters and German words) materials, and Shepherdson^31^ analyzed the data of two visual experiments with a two-alternative forced choice test. The presentation of the retro-cue increased drift rate compared to the no-cue condition, particularly in the visual tasks. This result is in line with the assumption that retro-cues strengthens and protects the representation of the cued item from subsequent visual interference^32–34^. Retro-cues also reduced nondecision time, and this effect was larger the more items had to be remembered. This finding was interpreted as indicative that retro-cues allowed a head start in the retrieval of the relevant representation before the decision process could take place^34^. Finally, retro-cues also had some unsystematic effects on boundary separation: in some experiments, boundary separation was reduced in retro-cue trials. This is suggestive that participants were sometimes less conservative in the presence of a retro-cue. So far, we are not aware of diffusion modeling attempts of the retro-cue effect in the delayed estimation paradigm. Accordingly, one aim of the current study was to assess if retro-cues exhibit consistent effects on diffusion model parameters for both change detection and delayed estimation paradigms using visual materials.

So far, studies have not examined age-related changes in focusing efficiency through the lens of the drift diffusion model. This can help us overcome issues related to age and individual differences in task approach, such as preferences to respond fast while sacrificing accuracy or to respond slowly but accurately (i.e., speed-accuracy tradeoffs).

### Individual differences in speed-accuracy tradeoffs and in focusing efficiency

Individuals can set different criteria to decide, with some favoring speed over accuracy whereas others favor accuracy over speed. Traditionally, speed and accuracy are treated separately when analyzing task performance which is not ideal for rank-ordering individuals in terms of their abilities. Diffusion modeling overcomes this issue by creating a single metric to compare individuals with regards to theoretically meaningful processing stages^35^. Accordingly, its parameters were shown to have separate relations to working memory capacity and reasoning^36–38^.

So far, studies have not evaluated whether the diffusion model can help reveal individual differences in focusing efficiency in working memory. In general, the literature is still incipient regarding individual differences in the retro-cue benefit. Robison and Unsworth^39^ observed a small but significant correlation between the retro-cue benefit obtained in a single task with working memory capacity measured with a battery of three tasks. Ye et al.^40^ observed a small correlation between the retro-cue benefit in two separate tasks, yet these did not correlate with performance in another independent working memory task. The use of a single task or a small sample size, however, precludes firm conclusions to be taken from these studies. Therefore, addressing individual differences in the retro-cue effect through diffusion modeling parameters was the final goal of the present study.

### The present study

The main goal of the present study was to model the data of retro-cue tasks to assess age differences in focusing efficiency in working memory while accounting for possible individual differences in speed-accuracy tradeoffs. We analyzed data of a large sample of younger (n = 172, mean age = 23.7 years) and older adults (n = 174, mean age = 71.5 years) in four retro-cue tasks (see Fig. 1) varying the type of retrieval paradigm (change-detection vs. delayed estimation), the material to be remembered (color vs. orientations), and the type of spatial cue (central arrows vs. peripheral circles). This is the largest dataset to date allowing both the assessment of age as well as individual differences in the retro-cue effect. We modeled the data using the EZ versions of the diffusion model for two-choice response tasks^25^ and for circular tasks^27^. This is the first time that the retro-effect in delayed estimation tasks is evaluated through this cognitive model, offering the opportunity to examine if retro-cues have similar effects in model parameters across different retrieval paradigms. Finally, this is also the first time that age and individual differences in the retro-cue effect are evaluated with the diffusion model, permitting an examination of which components of focused attention are more sensitive to age-related decline and the suitability of this effect as a valid psychometric indicator of focusing ability.

## Results

Table 1 presents descriptive statistics for the behavioral performance in each of the four retro-cue tasks and Bayes Factors (BF_10_) indicating the strength of evidence for or against the presence of a retro-cue effect (i.e., performance difference between No-Cue and Retro-Cue trials) in each age group, as well as for age differences in each condition separately. A BF_10_ > 1 indicates evidence in favor of the presence of an effect, and a BF_10_ < 1 indicate evidence against differences. We considered BF_10_ values between 0.3 and 3 as ambiguous, and values larger than 10 or smaller than 0.1 as showing strong support for or against an effect, respectively. Note that, for the delayed estimation paradigm, the measure of memory accuracy is circular variance since this reflects the average error in reporting the correct feature in the circular space. Accordingly, better performance in this paradigm implies lower values. Table 1 shows that retro-cue improved memory accuracy (i.e., increased proportion correct in change detection and reduced circular variance in delayed estimation tasks) and reduced reaction times (RTs) for all tasks and both age groups. In sum, retro-cues improved both response speed and accuracy for both age groups. The diffusion model analysis integrates over these two performance indicators. We will describe the modeling results next.

**Table 1:** Descriptive statistics for the two dependent variables in the four retro-cue tasks, and evidence (BF_10_) for condition effects (no-cue vs. retro-cue) and age effects (younger vs. older) in the baseline condition and condition effects.

Task	DV	Condition	Older		Younger		Age effect
			Mean	SD	Mean	SD	BF_10_
Color CD	Proportion Correct	No-Cue	0.76	0.10	0.78	0.10	0.21
		Retro-Cue	0.84	0.10	0.86	0.10	*0.05*
		BF_10_	**3.81 × 10**^**17**^		**6.93 × 10**^**15**^		
	Reaction Time (s)	No-Cue	1.57	0.33	1.00	0.22	**2.34 × 10**^**15**^
		Retro-Cue	1.14	0.28	0.65	0.18	2.75
		BF_10_	**4.14 × 10**^**17**^		**2.34 × 10**^**18**^		
Orientation CD	Proportion Correct	No-Cue	0.68	0.10	0.76	0.09	**1.80 × 10**^**18**^
		Retro-Cue	0.71	0.12	0.84	0.10	**9.35 × 10**^**16**^
		BF_10_	4.76		**9.86 × 10**^**14**^		
	Reaction Time (s)	No-Cue	1.85	0.44	1.28	0.28	**6.77 × 10**^**16**^
		Retro-Cue	1.36	0.38	0.86	0.21	*0.04*
		BF_10_	**7.06 × 10**^**15**^		**1.38 × 10**^**16**^		
Color DE	Circular Variance	No-Cue	28.24	13.61	22.74	11.53	**1.01 × 10**^**38**^
		Retro-Cue	14.20	11.97	9.07	8.47	**0.13**
		BF_10_	**2.05 × 10**^**19**^		**1.88 × 10**^**17**^		
	Reaction Time (s)	No-Cue	3.98	1.28	3.03	1.02	**1–08 × 10**^**26**^
		Retro-Cue	2.99	1.14	2.40	0.88	**4.15 × 10**^**17**^
		BF_10_	**3.26 × 10**^**14**^		**7.53 × 10**^**15**^		
Orientation DE	Circular Variance	No-Cue	25.29	12.39	24.19	12.35	0.26
		Retro-Cue	17.34	13.38	16.21	12.45	0.12
		BF_10_	**4.97 × 10**^**33**^		**3.62 × 10**^**27**^		
	Reaction Time (s)	No-Cue	2.76	0.77	2.09	0.59	**3.26 × 10**^**14**^
		Retro-Cue	2.30	0.74	1.49	0.49	**4.03 × 10**^**5**^
		BF_10_	**2.05 × 10**^**16**^		**9.39 × 10**^**14**^		

*Note*. CD = change-detection. DE = delayed estimation. N = number of participants with data to be included. Bayes Factors in bold indicate strong evidence in favor of an effect (BF_10_ > 10), Bayes Factors in italic strong evidence against an effect (BF_10_ < 0.1).

### Retro cue effects on diffusion model parameters

We calculated diffusion model parameters using the EZ-diffusion model^25^ for change detection paradigms and the EZ-Circular Diffusion model^27^ for the delayed estimation paradigms. To verify that both the models captured the observed data adequately, we generated synthetic data from the computed parameters for all participants in each task and correlated the observed data with the generated data (see Table 2 for a summary of these correlations). These correlations ranged from 0.73 to 0.98, indicating that the calculated parameters fit the observed data acceptably. Generally, model fit was better for the change detection than for the delayed estimation tasks. Yet, model fit was similar for both age groups as well as between conditions. Thus, both age and condition differences cannot be explained by differences in model fit. Plots illustrating the model fit and a more elaborate discussion of model fit are presented in the Online Supplementary Materials.

**Table 2:** Correlation between observed and model generated summary statistics to evaluate model fit of the EZ-DM and EZ-CDM.

Task	Domain	PC		RT Q25		RT Q50		RT Q75	
		Older	Younger	Older	Younger	Older	Younger	Older	Younger
CD	Color	.86	.87	.96	.98	.96	.98	.94	.95
	Orientation	.84	.86	.92	.96	.95	.97	.93	.96

		Circular variance		RT Q25		RT Q50		RT Q75	
		Older	Younger	Older	Younger	Older	Younger	Older	Younger
DE	Color	.95	.95	.87	.78	.86	.78	.83	.76
	Orientation	.92	.89	.82	.87	.81	.85	.73	.81

PC = Proportion correct; RT = Reaction time; Q = Quantile; CD = Change detection; DE = delayed estimation.

**Figure 3:**
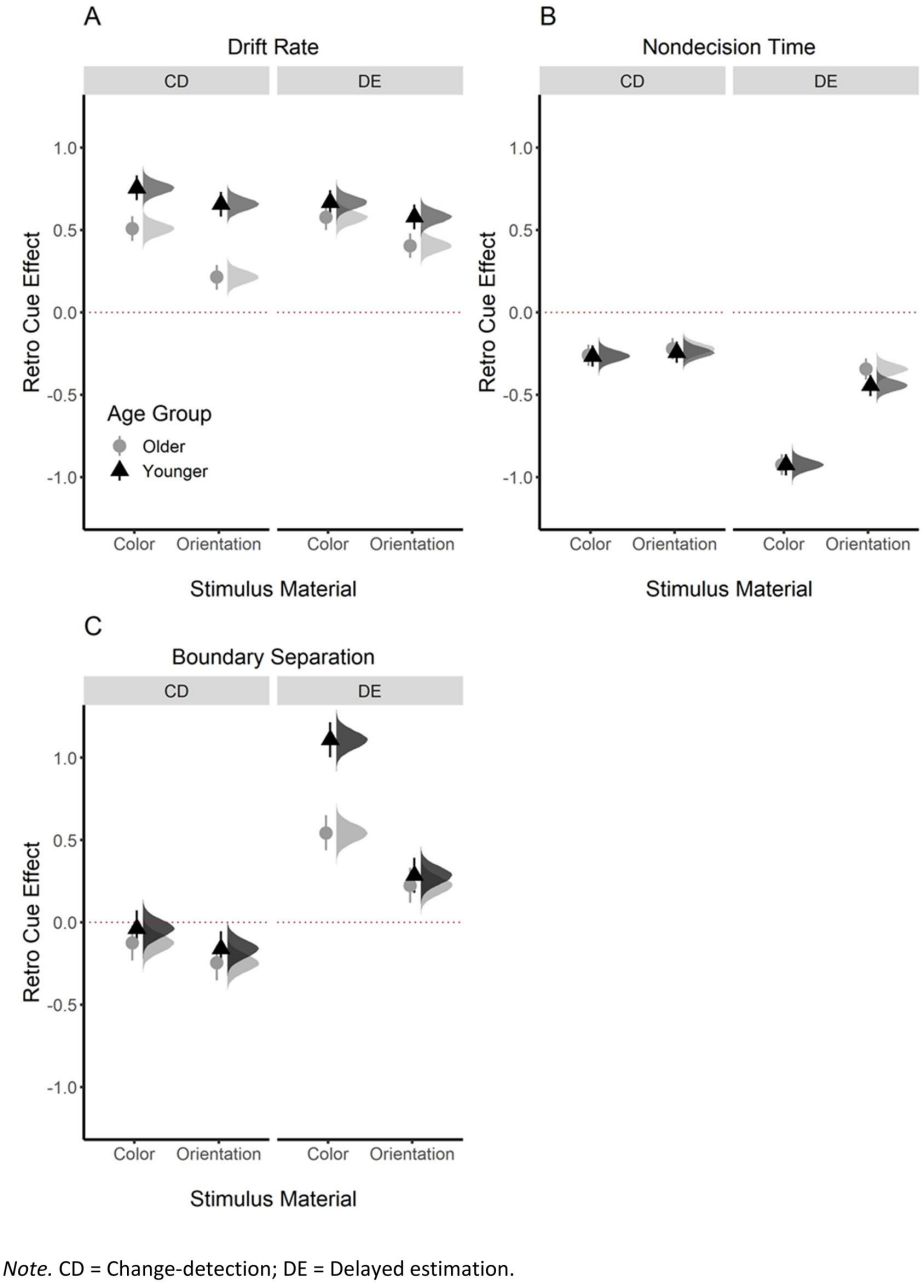
Posterior estimates (mean, 95% HDI, and full distribution) for the retro cue effect on drift rate (**A**), nondecision time (**B**), and boundary separation (**C**) for younger (black triangles) and older (grey circles) adults. CD = Change-detection; DE = Delayed estimation.

Next, we separately submitted drift rate, nondecision time, and boundary separation to a Bayesian linear mixed effects model estimating the parameter values for the no-cue condition and parameter changes yielded by the retro-cue for each age group and task. Figure 3 illustrates the posterior estimates of the retro-cue effect (i.e., difference between the no-cue and retro-cue condition) in each task and age group with regards to the drift rate (panel A), nondecision time (panel B), and boundary separation (panel C). In Table 3, we report the parameter mean and the interval covering 95% of its posterior distribution (aka. its highest density interval, HDI). We also calculated Bayes Factors in support of the presence of an effect (BF_10_) using the Savage-Dickey density method (see details in the Methods section). Additionally, Table 3 summarizes the results of mixed effect models for each age group, task, and diffusion model parameter, as well as the evidence (BF_10_) for retro-cue and age effects. Below, we will first report an analysis of the retro-cue effect averaged across age groups and focus on age differences later.

**Table 3:** Posterior Means and 95% Highest Density Intervals of the Diffusion Model Parameters for the No-Cue Condition and the Retro-Cue Effect in Each Task. Additionally, the Evidence (Bayes Factor, BF_10_) for Age Differences in the No-Cue Condition, and the Retro-Cue Effect for each Age Group are Displayed.

Task	Age group	No-Cue		Retro-cue effect		BF_10_ Retro cue effect
		Mean	[95% HDI]	Mean	[95% HDI]	
**Drift (ν)**						
Color CD	Younger	1.25	[1.15, 1.35]	0.75	[0.68, 0.83]	**1.56 × 10**^**18**^
	Older	0.86	[0.78, 0.93]	0.51	[0.43, 0.58]	**6.16 × 10**^**15**^
	BF_10_ age effect	**4.03 × 10**^**18**^		**4.39 × 10**^**3**^		
Orientation CD	Younger	0.91	[0.83, 0.99]	0.66	[0.58, 0.73]	**1.17 × 10**^**16**^
	Older	0.51	[0.45, 0.57]	0.21	[0.14, 0.29]	**3.16 × 10**^**15**^
	BF_10_ age effect	**7.22 × 10**^**15**^		**1.46 × 10**^**15**^		
Color DE	Younger	0.87	[0.81, 0.94]	0.67	[0.59, 0.74]	**2.49 × 10**^**15**^
	Older	0.62	[0.55, 0.69]	0.58	[0.50, 0.65]	**8.01 × 10**^**17**^
	BF_10_ age effect	**1.77 × 10**^**16**^		0.31		
Orientation DE	Younger	1.03	[0.95, 1.12]	0.58	[0.50, 0.66]	**5.15 × 10**^**20**^
	Older	0.81	[0.74, 0.88]	0.40	[0.33, 0.48]	**1.53 × 10**^**20**^
	BF_10_ age effect	**227.11**		8.49		
**Nondecision time (T**_**er**_**)**						
Color CD	Younger	0.64	[0.59, 0.68]	− 0.27	[− 0.33, − 0.20]	**1.69 × 10**^**54**^
	Older	0.96	[0.91, 1.01]	− 0.26	[− 0.33, − 0.20]	**3.44 × 10**^**20**^
	BF_10_ age effect	**1.15 × 10**^**164**^		*0.06*		
Orientation CD	Younger	0.75	[0.70, 0.79]	− 0.24	[− 0.31, − 0.18]	**2.03 × 10**^**22**^
	Older	1.00	[0.95, 1.04]	− 0.22	[− 0.29, − 0.16]	**2.96 × 10**^**18**^
	BF_10_ age effect	**3.20 × 10**^**21**^		*0.08*		
Color DE	Younger	1.29	[1.21, 1.37]	− 0.93	[− 0.99, − 0.86]	**3.27 × 10**^**16**^
	Older	1.67	[1.58, 1.76]	− 0.93	[− 0.99, − 0.86]	**5.83 × 10**^**16**^
	BF_10_ age effect	**9.84 × 10**^**16**^		*0.06*		
Orientation DE	Younger	0.91	[0.84, 0.97]	− 0.44	[− 0.51, − 0.38]	**3.11 × 10**^**23**^
	Older	1.04	[0.96, 1.12]	− 0.34	[− 0.41, − 0.28]	**6.41 × 10**^**52**^
	BF_10_ age effect	1.78		0.63		
**Boundary separation (*a*)**						
Color CD	Younger	1.13	[1.05, 1.20]	− 0.04	[− 0.14, 0.07]	0.14
	Older	1.48	[1.40, 1.55]	− 0.13	[− 0.23, − 0.02]	1.53
	BF_10_ age effect	**2.75 × 10**^**17**^		0.21		
Orientation CD	Younger	1.38	[1.31, 1.46]	− 0.16	[− 0.27, − 0.05]	8.33
	Older	1.67	[1.59, 1.74]	− 0.25	[− 0.35, − 0.14]	**2.17 × 10**^**5**^
	BF_10_ age effect	**4.51 × 10**^**17**^		0.19		
Color DE	Younger	1.92	[1.77, 2.06]	1.11	[1.00, 1.21]	**4.23 × 10**^**17**^
	Older	2.11	[1.98, 2.24]	0.54	[0.44, 0.65]	**8.32 × 10**^**15**^
	BF_10_ age effect	0.91		**5.12 × 10**^**15**^		
Orientation DE	Younger	1.52	[1.39, 1.65]	0.28	[0.18, 0.39]	**2.28 × 10**^**15**^
	Older	1.86	[1.77, 1.96]	0.22	[0.12, 0.33]	**4.27 × 10**^**4**^
	BF_10_ age effect	**3.83 × 10**^**3**^		0.15		

Bayes Factors in bold indicate strong evidence in favor of an effect (BF_10_ > 10), Bayes Factors in italic strong evidence against an effect (BF_10_ < 0.1).

#### Drift rate

Consistent with previous findings^30,31^, retro-cues increased drift rate, Δ = 0.54, HDI = [0.52, 0.57], BF_10_ = 1.96 × 10^15^ (Fig. 3A). There was moderate evidence for the benefit on drift rate being similar for the change detection and delayed estimation paradigms, Δ = − 0.02 [− 0.08, 0.03], BF_10_ = 0.11. However, drift rate benefits were larger for tasks using color than orientation as memoranda, Δ = 0.16 [0.11, 0.22], BF_10_ = 2.94 × 10^17^.

#### Nondecision time

In agreement with previous findings^30,31^, retro-cues reduced nondecision time, Δ = − 0.45 [− .48, − 0.43], BF_10_ = 3.54 × 10^16^ (Fig. 3B). Retro-cue benefits for nondecision time were larger in the delayed estimation than in the change detection paradigms, Δ = 0.41 [0.37, 0.46], BF_10_ = 7.55 × 10^19^, and they were larger for the tasks using color than orientation as memoranda, Δ = 0.28, [0.24, 0.33], BF_10_ = 6.07 × 10^22^. As evident from Fig. 3B and Table 3, these differences were mainly driven by the very large retro-cue effect in the color delayed estimation task.

#### Boundary separation

For boundary separation, retro cue benefits were less consistent across the four tasks (Fig. 3C). In the change detection paradigm, there was evidence for a decrease in boundary separation in retro cues trials, Δ = − 0.14 [− 0.20, − 0.09], BF_10_ = 2.25 × 10^18^. The effect was small and ambiguous in the color task, Δ = − 0.08 [− 0.16, − 0.01], BF_10_ = 1.10, but stronger and well-supported in the orientation task, Δ = − 0.20 [− 0.28, − 0.13], BF_10_ = 2.35 × 10^25^. This varied pattern has also been observed previously in the literature^30^. In the delayed estimation paradigm, boundary separation increased consistently in retro-cue trials, Δ = 0.54 [0.49, 0.59], BF_10_ = 1.23 × 10^17^. This was the case both for the color, Δ = 0.83, [0.75, 0.90], BF_10_ = 1.15 × 10^26^, and orientation versions, Δ = 0.25 [0.18, 0.33], BF_10_ = 9.40 × 10^15^.

### Age differences in the no-cue condition and in the retro-cue benefit

**Figure 4:**
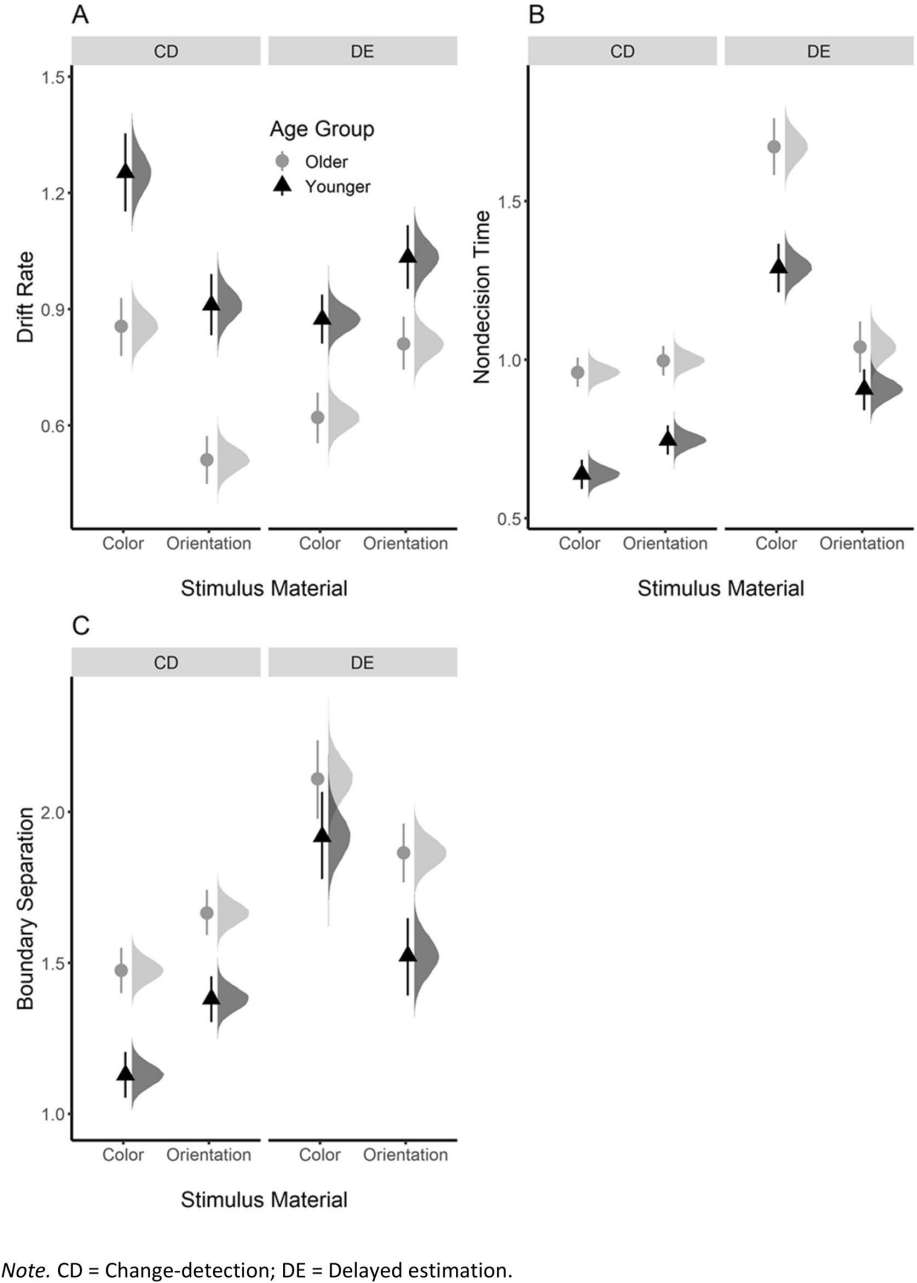
Posterior estimates (mean, 95% HDI, and full distribution) of the drift rate (**A**), nondecision time (**B**), and boundary separation (**C**) in the no-cue condition for younger (black triangles) and older (grey circles) adults in each task. *Note*. CD = Change-detection; DE = Delayed estimation.

Our second aim was to assess age differences in the size of the retro-cue effects across the diffusion model parameters. However, because age groups also differed in baseline performance (i.e., in the no-cue condition), we will briefly present the source of these differences first, followed by the assessment of the retro-cue effect. Figure 4 shows the posterior estimates of the three diffusion model parameters in the no-cue condition for younger and older adults in each task. Table 3 provides the BF for the age comparisons.

#### Drift rate

*No-cue condition* Consistent with prior results^41,42^, younger adults showed higher drift rates in the no-cue condition than older adults, Δ = 0.32 [0.26, 0.37], BF_10_ = 1.45 × 10^23^ (Fig. 4A). There was moderate evidence for this age difference being larger in change detection than delayed estimation, Δ = 0.16 [0.05, 0.27], BF_10_ = 5.09; but evidence against an effect of material (i.e., color vs. orientation), Δ = 0.01 [− 0.09, 0.12], BF_10_ = 0.007.

*Retro-cue benefit* Younger adults showed larger retro-cue benefits on drift rates than older adults, Δ = 0.24 [0.19, 0.29], BF_10_ = 6.60 × 10^15^ (Fig. 3A). There was strong evidence for this age difference being larger in the change detection than delayed estimation paradigms, Δ = 0.21 [0.11, 0.32], BF_10_ = 120. Age differences were descriptively larger in the color than orientation task versions, Δ = − 0.14 [− 0.25, − 0.03], but the evidence for this effect was ambiguous, BF_10_ = 2.36.

#### Nondecision time

*No-cue condition* In line with previous results^41^, nondecision time in the no-cue condition was lower for younger than for older adults, Δ = − 0.27 [− 0.32, − 0.23], BF_10_ = 1.55 × 10^20^ (Fig. 4B). There was no effect of paradigm, Δ = − 0.03 [− 0.12, 0.06], BF_10_ = 0.008, but age differences were larger for the color than orientation material, Δ = − 0.16 [− 0.25, − 0.07], BF_10_ = 19.80.

*Retro-cue effect* There was moderate evidence against age differences in the retro-cue effect on nondecision time, Δ = − 0.03 [− 0.08, 0.01], BF_10_ = 0.10. As displayed in Table 3, there was strong evidence against age differences in nondecision time for all tasks, all BFs_10_ < 0.10, except the orientation delayed estimation task, BF_10_ = 0.63. All in all, these results indicate that there are no or only negligible age differences in the retro-cue effect on this parameter.

#### Boundary separation

*No-cue condition* In agreement with prior results^41^, boundary separation was lower for younger than older adults in the no-cue condition, Δ = − 0.29 [− 0.36, − 0.22], BF_10_ = 2.49 × 10^141^ (Fig. 4C). There was moderate evidence against an effect of paradigm, Δ = − 0.05 [− 0.20, 0.10], BF_10_ = 0.12, and material (color vs. orientation), Δ = 0.05 [− 0.10, 0.19], BF_10_ = 0.12.

*Retro-cue effect* As retro-cue effects differed considerably between the change detection and delayed estimation paradigms, we did not calculate age differences across all tasks. In the change detection paradigm, retro-cue effects were descriptively larger for older than for younger adults, Δ = 0.09 [− 0.02, 0.19], however this difference was not supported statistically, BF_10_ = 0.39. In the delayed estimation paradigm, there was strong evidence for a larger retro-cue effect on boundary separation in younger than for older adults, Δ = 0.31 [0.21, 0.42], BF_10_ = 1.03 × 10^17^. Yet, as shown in Fig. 3C and Table 3, this effect was mainly driven by the color delayed estimation task.

In sum, our results indicate a single source of age-related decline on retro-cue benefits, namely on the drift rate parameter. Effects were absent or negligible in nondecision time, and inconsistent on boundary separation.

### Are there consistent individual differences in the retro-cue benefit?

**Figure 5:**
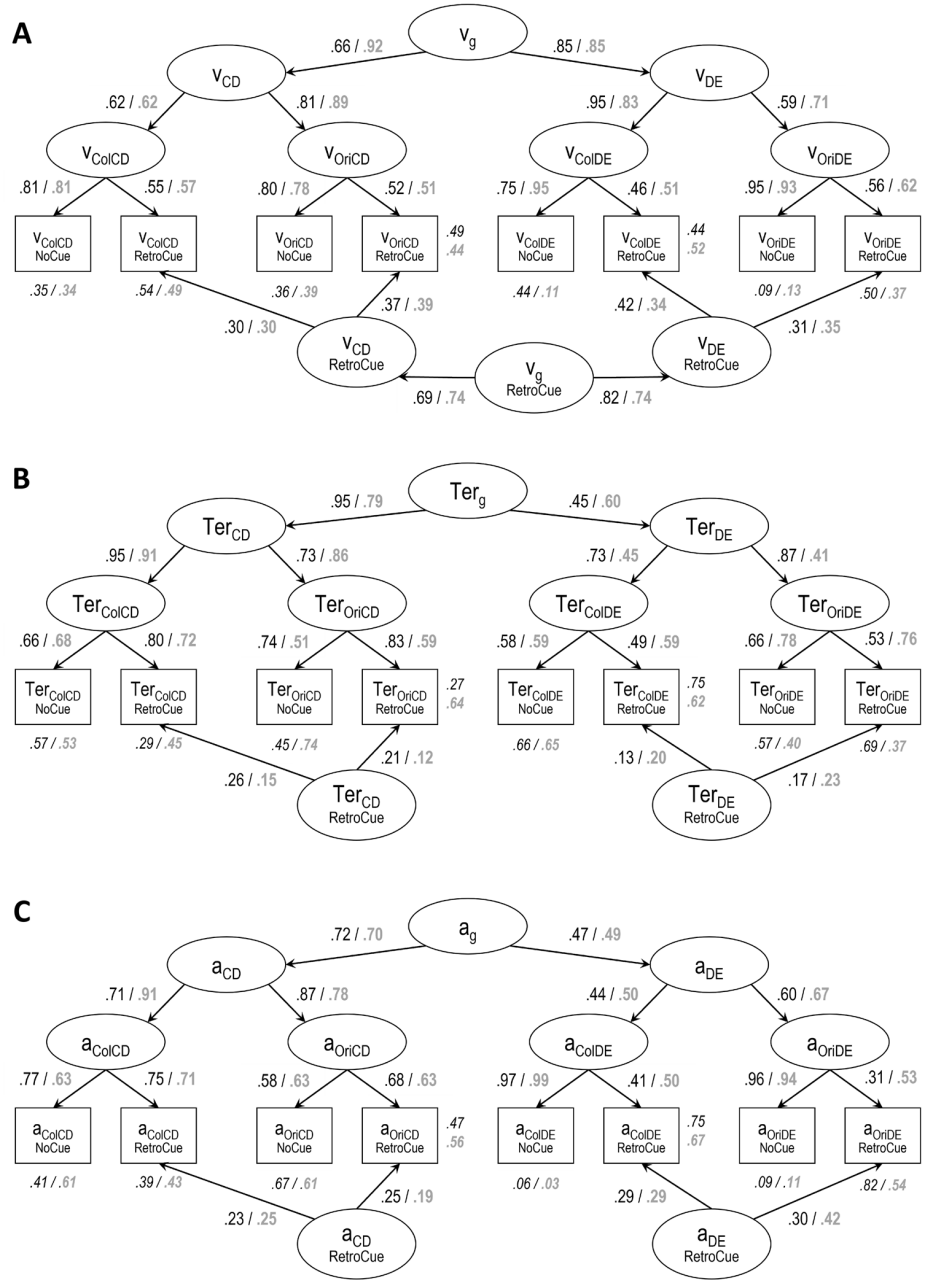
Simplified path diagrams for the bayesian structural equation models isolating condition general variance from variance specific to the ability to focus attention (i.e., benefitting from retro-cues) for drift rate (**A**), nondecision time (**B**), and boundary separation (**C**). We report posterior means of standardized path coefficients for younger (black font) and older (grey font) adults. Additionally, posterior means of the error variances for each indicator are displayed in italics. *Note* v = drift rate, Ter = nondecision time, a = boundary separation. Col = color, Ori = orientation, CD = change detection, DE = delayed estimation, g = general.

Our third research question focused on individual differences in the retro-cue effect. Our goal was to determine whether we can estimate a paradigm- and material-general retro-cue factor. To address this question, we estimated Bayesian structural equation models (BSEM, for details see the Methods section) separating general variance shared between the no-cue and the retro-cue conditions from general variance driven only by the retro-cue. Additionally, we accounted for retrieval paradigm- and material-specific variance using a hierarchical factor model. Figure 5 presents the path diagrams of the BSEM for drift rate, non-decision time, and boundary separation, and Table 4 presents the posterior estimates for the proportion of variance explained by the different latent variables for each indicator.

**Table 4:**
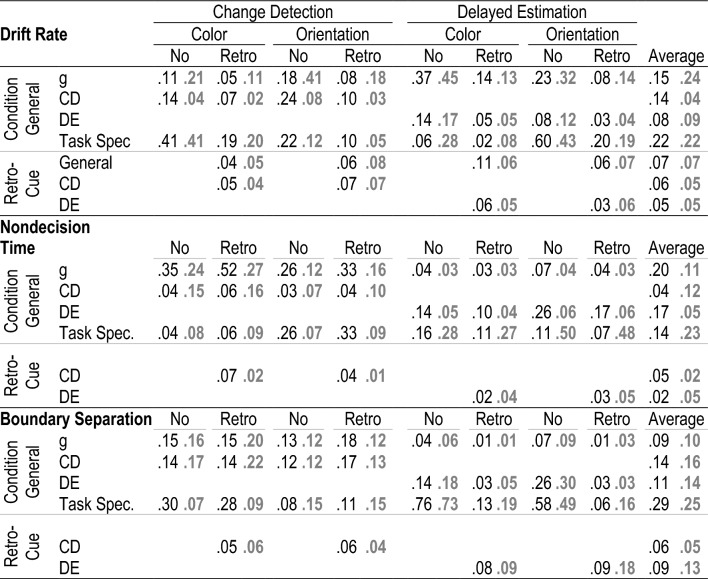
Posterior means of the proportion of variance in indicators explained by the different latent factors (i.e., determination coefficients) for younger (black) and older (bold, grey) adults.

G = general; CD = Change detection; DE = delayed estimation; Task Spec. = task specific.

#### Drift rate

The BSEM modeling individual differences in drift rates fit well to the data, PPP = 0.256, BCFI = 0.985 [0.965; 1.000], BRMSEA = 0.041 [0.000, 0.070]. There were consistent retro-cue general individual differences in drift rates (ν_g_ retro-cue)—on average, this factor explained 7% of total variance in indicators for both older and younger adults. However, the general factor (i.e., ν_g_) capturing variance shared across conditions contributed more strongly to individual differences in drift rate—15% and 24% for younger and older adults, respectively. Additionally, the factors isolating paradigm-specific variance (i.e., change detection and delayed estimation) captured, on average, 8 to 14% of variance in indicators for younger and 4 to 9% of variance for older adults. Task specific factors explained, on average, 22% of variance for both age groups.

In sum, retro-cue general individual differences were observed in drift rate. Yet, most of the individual differences in this parameter were condition general and therefore not driven specifically by focused attention efficiency.

#### Nondecision time

For nondecision time, the SEM model fitted the data well, BCFI = 0.976 [0.940; 1.000] and BRMSEA = 0.035 [0.000, 0.063]. Unlike drift rates, there were less consistent individual differences in the retro-cue benefit for nondecision time. In fact, we omitted a general factor of retro-cue benefits (i.e., there is no Ter_g_ retro-cue factor in Fig. 5B) without compromising model fit, indicating that there were only paradigm-specific contributions to the retro-cue benefit (see Table 4). For change detection, 5% and 2% of variance was explained by the change-detection retro-cue factor (Ter_CD_ retro-cue) for younger and older adults, respectively. For delayed estimation (Ter_DE_ retro-cue), these values were 2% and 5% for younger and older adults, respectively. Thus, mainly condition general factors captured individual differences. For example, the paradigm- and material-general factors for individual differences in non-decision time (i.e., Ter_g_) captured on average 20% of variance in indicators for younger and 11% of indicator variance for older adults.

#### Boundary separation

The SEM model for boundary separation had an acceptable fit to the data, BCFI = 0.930 [0.891; 0.967], BRMSEA = 0.073 [0.052, 0.093]. Including a general factor retro-cue factor for boundary did not improve model fit and indicated negligible shared variance between retro cue benefits in the change detection and delayed estimation tasks. So, like for nondecision time, there were only paradigm specific individual differences in the retro-cue benefit. The change detection retro-cue factor (i.e., a_CD_ retro-cue) captured, on average, 6% of variance in indicators for younger and 5% for older adults. The delayed estimation retro-cue factor (i.e., a_DE_ retro-cue) explained, on average, 9% of variance in indicators for younger and 13% for older adults. For boundary separation, most individual differences were captured by task-specific factors: on average, 29% of indicator variance for younger and 29% for older adults. In contrast, the general factor shared across all tasks and conditions (i.e., a_g_) only captured 9% of variance in indicators for younger and 10% for older adults. This indicates that there is primarily task specific variance in boundary separation.

## Discussion

Our main aim was to assess for an age-related attention deficit in working memory^43^ using the retro-cue paradigm as a testbed. So far, there were mixed findings regarding age-related impairments in the retro-cue effect. Here, we reasoned whether these discrepant findings were related to the diversity of task paradigms used across studies (i.e., change-detection vs. delayed estimation) or to task-specific strategies (e.g., setting of speed-accuracy tradeoffs). To address these issues, we submitted to a drift diffusion model a large dataset of younger and older adults that completed four retro-cue tasks. This allowed us to examine the retro-cue effect through changes in model parameters that integrate speed and accuracy measures. Younger and older adults benefited from retro-cues and these benefits accrued in similar model parameters for both age groups across different memory paradigms. We observed only one source of age-related decline: older adults had a smaller attentional boost on evidence accumulation (aka. drift rate). Next, we will discuss first the meaning of the retro-cue effects on the different diffusion parameters, followed by a discussion of the meaning of age-related changes in these parameters.

### Retro-cue effects in diffusion model parameters

Replicating prior literature^30,31^, retro-cues reliably increased drift rate and reduced nondecision time. To the best of our knowledge, this is the first time the retro-cue effect in the delayed estimation paradigm is examined through the diffusion model. Our results suggest that both modeling frameworks (for two choices and for circular choices) are similarly sensitive to the experimental effects of retro-cues.

The retro-cue benefit in drift rate has been explained as reflecting the strengthening and protection of the cued item from subsequent visual interference^30,31^: this protection is irrelevant in verbal tasks (for which no drift rate benefit was observed in previous studies) and it does not undo the damage imposed by high levels of memory load. Conversely, the retro-cue effect in the nondecision time parameter was interpreted as indicative that participants use it for the advanced retrieval of the target representation into the focus of attention ahead of the test^34^. This allows a well retrieved representation to enter the decision, reducing inter-item competition in memory, and consequently the impact of memory load.

The effects of retro-cues on boundary separation were less consistent. In the change-detection paradigm, a credible effect was observed in the orientation but not the color version. This mixed pattern is similar to the inconsistent effects of retro-cues on boundary observed by Shepherdson and collaborators^30,31^. If present, the reduction in boundary separation in change detection indicates that participants were sometimes less conservative in retro-cue than in no-cue trials. For the delayed estimation paradigm, conversely, retro-cues increased boundary separation, indicating higher response conservativeness. This divide could be explained as follows. In delayed estimation, it takes time to adjust the probe feature to closely match the remembered information, whereas in change detection memory precision and motor control requirements are low. Accordingly, RTs are much slower in delayed estimation than change detection. Although retro-cues also reduced RTs in delayed estimation, this benefit was not proportional to the accuracy benefit. Accuracy improvements in delayed estimation were observed with a reward manipulation, and this effect was accompanied by the slowing of RTs^44^. This was interpreted as evidence that retrieval is costly, and motivation is needed to engage with retrieving a more precise representation. Hence retro-cues might have two effects in delayed estimation. On one hand, they protect memory representations from interference, increasing drift rate which reduces RTs. On the other hand, as the mental representation is more precise, participants are motivated to give a precise response, so they increase response criterion which slows RTs.

### How age impacted the retro-cue effect in diffusion model parameters?

Our older adults showed, in general, lower drift rates, higher nondecision time, and a higher boundary separation than younger adults in the no-cue (baseline) condition. These findings replicate prior results indicating lower working memory capacity in aging^41^. Here we focused on how attention improved these parameters. For nondecision time and boundary separation, younger and older adults benefited similarly from retro-cues. For nondecision time, this indicates preserved efficiency in using the cue for a head start on the retrieval of the relevant representation, whereas for boundary separation, it suggests similar motivation changes yielded by the retro-cue.

The only parameter that showed age-related decline in focused attention was the drift-rate. Older adults had credibly smaller benefits in three of the four tasks. This novel result puts into perspective the findings from prior studies reporting preserved focusing ability in aging^13–17^. These prior findings were mainly based on memory accuracy measures. In fact, when analyzing only this indicator, we have also mostly observed evidence for preserved ability^45^. By integrating between accuracy and RTs, the diffusion modeling revealed that older adults do not gain as much from retro-cues as younger adults in terms of the quality of the information entering the decision process. This suggests an aging deficit in using focused attention to effectively strengthen and protect mental representations from interference. This is consistent with results by Loaiza and Souza^46^. In their study, younger adults (but not the older ones) maintained the retro-cued item protected from a subsequent distractor task. This suggests that older adults have difficulties managing interference. One may wonder whether these results could be explained by older adults being more prone to time-based forgetting given that the retro-cue trials took overall longer to complete. However, prior studies have not observed more time-based forgetting in older adults^14,47^. Future studies should therefore target age-related changes in the strengthening and protection of memory representations against interference and consider both accuracy and speed measures to get a complete picture of how older adults approach the task, and how they use information to make decisions.

### Sources of individual differences in focusing efficiency

Our findings also have implications for the assessment of individual differences in focusing efficiency. Our results indicate that only drift rate captured individual differences in retro-cue benefits that are shared between paradigms and materials, whereas nondecision time and boundary separation showed only paradigm-specific sources of variance. Additionally, individual differences in the retro-cue benefit were much smaller than individual differences shared across experimental conditions, corroborating previous reports of small and unreliable individual differences in experimental effects^48,49^. They also underscore the importance of using several tasks and paradigms to extract general individual differences^36,51,52^.

The robust age and individual differences in focusing efficiency on drift rates is consistent with prior findings relating this parameter to other higher cognitive functions such as working memory capacity and reasoning ability^36,37,50^. Hence, our findings corroborate drift rate as the most psychometrically relevant diffusion parameter.

## Conclusion

Overall, using diffusion modeling to examine the effects of retro-cues seems as a promising venue to identify sources of age and individual differences. Our findings indicate that, although small, the retro-cue effect in drift rate could be taken as valid psychometric indicator of focusing efficiency: people reliably differ in their ability to use focused attention to strengthen and protect a memory representation in working memory, and this ability is prone to age-related decline.

## Method

### Participants

Participation criteria were: (a) age between 18 and 35 years old (younger sample) or between 65 and 80 (older sample), (b) fluent in German, and (c) physically and mentally healthy as evaluated by self-report, and for the older adults, a score higher than 25 in the Mini-Mental State Examination^53^. We advertised the study in a high circulation magazine in the German-speaking part of Switzerland. Most of the older participants were recruited this way. Younger adults were students from the Zurich area. Participants were offered 15 Swiss francs (ca. 16 dollars) per hour of participation, or in the case of students, they could also opt for receiving partial course credits. Participants signed an informed consent form at the beginning of the study and were debriefed at the end. The study protocol was conducted in accordance with the Declaration of Helsinki (excepting study registration) and all relevant ethical regulations, and it was approved by the Institutional Review Board of the Psychology Institute of the University of Zurich (approval number 16.12.12),.

We aimed to achieve a sample size of at least 150 participants in each age group. Our final sample size consisted of 172 younger adults (*M* = 23.7 years old, *SD* = 3.81; 133 women) and 174 older adults (*M* = 71.5 years old, *SD* = 4.3; 97 women).

The data reported in this paper is part of a large battery of cognitive tasks. Part of the data was reported in Souza et al.^45^. The study consisted of two laboratory sessions, each lasting between 2.5 and 4.5 h. Older adults took in general longer to complete the tasks since they were not time-limited. In total, participants completed 20 tasks that were evenly distributed across sessions. The tasks measured working memory capacity (n = 2), reasoning (n = 3), perceptual ability (n = 3), multiple object tracking (n = 1), and attentional selection with regard to space (n = 3), features (n = 4), and working memory contents (n = 4). Only the four working memory attentional selection tasks (aka retro-cue tasks) will be reported here. Sessions occurred in a group lab in which up to five people could be tested simultaneously. The computer stations were arranged in a row with dividers between them. Two large 10-min breaks were scheduled per session. Participants were offered drinks (tea, coffee, water, juice) and snacks (cake, cookies, chocolate, fruit, nuts) during the large breaks.

### Stimuli and procedure

#### Difficulty calibration

Capacity limitation in working memory affects both how information is stored (its precision) as well as how evidence is processed to reach a decision. Guest et al.^42^ showed that age differences in evidence accumulation and asymptotic performance vary as a function of memory load. Younger adults showed a higher asymptotic performance than older adults when maintaining one or two items in working memory indicating that they stored information with higher precision. Yet, evidence accumulation only showed age-related decline when memory load was larger than one, indicating that older adults have difficulty in deciding when multiple memory elements are available. This suggests that it is important to control for memory load across age groups, such that we can separate difficulties in maintaining memory representations from memory selection afforded by the retro-cue. Here we attempted to maintain task difficulty at similar levels across age groups by calibrating memory load.

We ran a pilot study to determine the memory load of the working memory for each age group. In the pilot study, younger (n = 30) and older (n = 30) adults completed a version of the retro-cue tasks in which the memory load was individually adjusted using a staircase procedure (QUEST) to yield 75% accuracy in the no-cue condition in the change-detection tasks and a 40° recall error in the delayed estimation tasks. We used the average value of the memory load obtained in this pilot to determine the parameter value for each age group in the final study.

#### Feedback

For the change detection tasks, feedback was provided by presenting the German words for “correct” (Richtig) and “incorrect” (Falsch) in green and red, respectively, in the middle of the screen. For the delayed estimation tasks, feedback was presented by indicating the match between the response and the true target value. Trials were computer-paced, but small self-paced pauses were allowed every ten trials.

#### Retro-cue tasks

In all tasks, participants were not given specific instructions regarding how to set their speed or accuracy priorities. They were simply told how to respond on the task (e.g., indicate whether the probe match or mismatches; select the feature value using the continuous scale).

*Color change-detection* The task procedure is illustrated in Fig. 1A. In the beginning of a trial, a white fixation cross was shown against a grey background for 1000 ms. Next, a set of colored dots was presented simultaneously for 1000 ms. The memory items (radius = 32 pixels) were presented equally spaced around an imaginary circle centered in the middle of the screen (radius of the circle = 150 pixels). The number of items in the memory array (i.e., memory load) was defined for each age group separately (younger = 6 items; older = 5 items). Colors were sampled without replacement from twelve values: black [RGB: 0, 0, 0], brown [127, 45, 0], dark green [0, 63, 0], green [0, 255, 0], turquoise [90, 160, 255], blue [0,0,255], lilac [138, 20, 236], pink [255, 50, 255], red [255, 0, 0], orange [255, 127, 0], yellow [205, 205, 0], and beige [165, 141, 99]. In 50% of the trials, after a retention interval of 1000 ms, a retro-cue (central arrow) indicated the location of the to-be-tested item for 250 ms. After another 1000 ms blank post-cue interval, the test display appeared. In no-cue trials (50% of the trials), the test display appeared directly at the end of the 1000-ms retention interval.

At test, a single colored circle (probe) appeared in one of the locations previously occupied by a memory item. Participants had to indicate whether the probe color matched (50%, right-arrow keypress) or mismatched (50%, left-arrow keypress) the color of the item presented at that location. A non-matching color had either not been presented in the memory array (new probe; 25%) or it was presented in another location (intrusion probe; 25%). The probe was shown until a response was registered. Next, response feedback appeared for 1000 ms, and the subsequent trial started after a 500 ms blank interval. Participants completed four practice trials and 80 test trials. No-cue and retro-cue trials were randomly intermixed. To obtain parameters of the diffusion model we calculated the average proportion of correct responses, and the median and variance of correct RTs.

*Orientation change-detection* The task procedure is illustrated in Fig. 1B. This task was modelled after the one reported by Fougnie et al.^54^. The structure, timing, testing, dependent variable, feedback and number of trials in this task was as described for the color change-detection task. The orientation and color versions differed only in terms of the memoranda and the type of retro-cue. Regarding the memoranda: participants had to encode the orientation of a set of white isosceles triangles (radius = 100 pixels) presented equally spaced around an imaginary circle (radius = 200 pixels) centered in the middle of the screen. The memory load was set to each age group separately (younger = 5.4 items; older = 4.6 items; the non-integer values reflect the mixing of two values: for example, 5.4 items indicate that 60% of the trials had 5 items and 40%, 6 items). The orientation of the memory items was sampled from 8 values (45°, 90°, 135°, 180°, 225°, 270°, 315, or 360°). Regarding the retro-cue, it consisted of a white circle (radius = 60 pixels) that appeared at the position of the to-be-tested item for 250 ms. We opted for a peripheral cue to avoid the presentation of an arrow which also contains orientation information and hence could interfere with the memoranda. Like for the color change detection task, we used the average proportion correct, the median and variance of correct RTs to obtain parameters of the diffusion model.

*Color delayed estimation* The task procedure is illustrated in Fig. 1C. This task was identical to the color change-detection task, with five exceptions: (a) the memoranda consisted of continuously varying colors given by the 360 angular degrees in a color circle defined in the CIELAB space with L = 70, a = 20, b = 38, and radius = 60^55^, (b) memory load was adjusted to this specific task having as criterion a recall error of 40° (younger = 5.8 items; older = 4.5 items), (c) the memory test required the reproduction of the color of one memory item using a continuous color wheel, (d) there was visual feedback regarding the distance of the response to the correct color, and (e) the number of test trials was 100.

Memory colors were sampled in each trial without replacement from the 360 values. At test, a color wheel appeared surrounding all locations previously occupied by the memory items. Participants moved the mouse around the wheel to adjust the color of the probe item, and they confirmed their selection with a left-mouse click. Then visual feedback was displayed for 2000 ms: the color selected by the participant was marked with a small white circle on the wheel, and the correct color was marked with the green outline of a circle. A new trial started 500 ms thereafter. To estimate parameters of the circular diffusion model we calculated the average circular deviation, the circular variance, and the median and variance of RTs using the normalized interquartile range.

*Orientation delayed estimation* This task differed from the Orientation Change-Detection regarding five aspects: (a) the orientation of the memory items was sampled from any value from 1–360°, (b) memory load was adjusted separately for each age group (younger = 6 items; older = 4 items) having a criterion of 40° of recall error, (c) the memory test required the continuous reproduction of the remembered orientation, (d) response feedback included the presentation of the correct tested feature, and (e) the number of test trials was 100 (instead of 80).

During the memory test in this task, a randomly rotated white triangle (probe) appeared in one of the locations previously occupied by a memory item. Participants adjusted the probe orientation by rotating it using the mouse and confirmed their response with a left-mouse click. Next, visual response feedback was provided for 2000 ms: The participant’s response was displayed as a white filled triangle, and the correct orientation of the memory item was displayed as a superimposed green triangle outline. The subsequent trial started after a 500 ms blank interval. Akin to the color delayed estimation task, we used the average circular deviation, the circular variance, and the median and variance of RTs using the normalized interquartile range to calculate parameters of the circular diffusion model.

### Open resources availability

The anonymized data for all tasks reported here are available at the Open Science Framework at https://osf.io/sfycz. The analyses were implemented in R and the analysis scripts are also available on the OSF page.

### Data analysis

#### Preprocessing

The dependent variables of interest were the mean and variance of the RTs and the memory accuracy in each experimental condition (no-cue vs. retro-cue) of each task. For the change-detection tasks, we only considered the RTs for correctly answered responses. As indicators of memory accuracy, we computed the proportion of correct responses in the change-detection tasks, and the average deviance and circular variance of response angle and true target’s angle for the delayed estimation tasks.

To reduce the impact of RT outliers, we trimmed RTs as follows. For the change detection tasks, we used only RTs from correct responses and removed RTs faster than 100 ms and longer than 7.5 s. For the delayed estimation task, we removed RTs faster than 250 ms and longer than 15 s. Additionally, for both types of tasks, we removed intra-individual outlier RTs three standard deviations above or below the individual condition mean.

#### EZ-diffusion model fitting

For the change-detection paradigms, we calculated the EZ-Diffusion parameters for each participant in each condition (i.e., no-cue and retro-cue) for each task separately using the equations for robust estimation given in Wagenmakers et al.^26^. Fitting of the EZ-Diffusion model requires the mean and variance of RTs on correctly answered trials and the proportion of correct responses. Accuracies with values of 0 or 0.5 were corrected by adding to the proportion correct the value given by 1/(2*n), with n representing the number of trials. Accuracy values equal to 1 were reduced by removing 1/(2*n) from the proportion correct.

For fitting the EZ-Circular Diffusion model, we used the equations given by Qarehdaghi and Rad^27^. Akin to the change-detection tasks, we fitted the data of each participant in each condition of the two delayed estimation tasks separately. Fitting this model requires the mean and variance of all RTs (given that in this task, the segregation of correct and incorrect responses is not straightforward) and mean deviation as well as the circular variance of the response feature with regards to the true target feature. All analyses were implemented in R, and the scripts for fitting the models are available in our OSF page.

#### Assessing retro-cue effects and age-related changes

For assessing retro-cue effects and age effects therein, we implemented a Bayesian hierarchical generalized mixed effects regression model (BGLM) on each parameter of the diffusion model separately. We included age group, task paradigm, memory feature, and cue condition as predictors in the model, and we included random slopes for the effects of task and memory feature. The models were fitted using the *brms* package^56^ implemented in R^57^. For all models we used a normal prior with mean = 0, and SD = 0.5. Parameters were estimated with four MCMC chains, each containing 2000 warmup and 10,000 post-warmup samples. To evaluate convergence of the chains, we checked that the R-hat values were below 1.05.

We calculated Bayes Factors to quantify the support for the presence of an effect or age differences (BF_10_) using the Savage-Dickey density method^58^. For this, we obtained the full posterior of the estimate of the relevant experimental effect or age difference and compared it to the prior density. Specifically, the BF reflects the ratio of prior to posterior likelihood for the effect at a given constraint, usually zero for testing the evidence for or against an effect. Thus, a BF_10_ > 1 indicates evidence in favor of the presence of an effect, and a BF_10_ < 1 indicate evidence against differences. Following recommendations by^59^, we considered BF_10_ values between 0.3 and 3 as ambiguous, and values larger than 10 or smaller than 0.1 as showing strong support for or against an effect, respectively. Despite the large number of posterior samples that were used for the estimation of the Bayes Factor, the reported BFs are not perfectly stable, especially for very large values. Thus, BF reproduced when re-running the scripts shared online can slightly diverge from the ones reported in the manuscript.

#### Assessing individual differences

To assess individual differences in the retro-cue benefit we estimated Bayesian Structural Equation Models (BSEM) separating individual differences shared across experimental conditions from individual differences specific to the retro-cue conditions for each of the three diffusion model parameters—drift rate, nondecision time, and boundary separation. The BSEM were fit using the *blavaan* package^60^ implemented in R. Parameters were estimated using a multigroup specification without constraints across the two age groups. We used the default sampling procedure using STAN implemented in *blavaan* with six MCMC chains, each containing 2000 warmup and 5000 post-warmup samples. We ensured that parameter estimates converged by assessing that all R-hat values were below 1.05. We evaluated model fit using Bayesian versions of the comparative fit index and the root-mean square error of approximation^61^. For these we also report the 95% highest density interval based on the full posterior of the model estimation.

To assess the contribution of different latent factors to the total variation in indicators we computed determination coefficients as the ratio of variance of exogeneous factors or residual variance for endogenous factors to the total indicator variance. These determination coefficients can be interpreted in terms of proportion of variance of an indicator that is explained by one of the latent factors.

### Supplementary Information

Supplementary Information.

## Data Availability

Materials, Data, and Analysis Scripts are available at: https://osf.io/sfycz.
