# Supplementary material for: A diffusion model analysis of age and individual differences in the retro-cue benefit
Source: Sci Rep. 2023 Oct 13;13:17356. doi: 10.1038/s41598-023-44080-z (PMC10575881; doi:10.1038/s41598-023-44080-z)
Supplement: Supplementary file 1 — Supplementary Information. [file 41598_2023_44080_MOESM1_ESM.docx]

# Online Supplementary Materials

Souza, A. S., & Frischkorn, G. A Diffusion Model Analysis of Age and Individual Differences in the Retro-Cue Benefit

**Table of Contents**

[Online Supplementary Materials 1](#_Toc137140961)

[Model Fits 2](#_Toc137140962)

[Figure S1 3](#_Toc137140963)

[Scatterplots Relating the Observed Data and the Synthetic Data Generated based on the Computed Diffusion Model Parameters for the Change-Detection Tasks. The Diagonal Gray Line Indicates Perfect Fit. Different Colors Mark the Different Materials (Color vs. Orientation), and the Different Shapes the two Experimental Conditions (No-Cue vs. Retro-Cue). MeanPC = Mean Proportion Correct, q05 to q95 = 5 to 95 Percent Quantile of Reaction Times. 3](#_Toc137140964)

[Figure S2 4](#_Toc137140965)

[Scatterplots Relating the Observed Data and the Synthetic Data Generated based on the Computed Diffusion Model Parameters for the Delayed Estimation Tasks. The Diagonal Gray Line Indicates Perfect Fit. Different Colors Mark the Different Materials (Color vs. Orientation), and the Different Shapes the two Experimental Conditions (No-Cue vs. Retro-Cue). MeanPC = Mean Proportion Correct, q05 to q95 = 5 to 95 Percent Quantile of Reaction Times. 4](#_Toc137140966)

## Model Fits

To assess model fit, we generated synthetic data from the parameters computed via the EZ-diffusion and the EZ-circular diffusion model. Then we calculated the correlation of the generated data with the observed data in each task and dependent variable (see Table 2 in the manuscript). The correlations capture the rank stability in the model parameters. Here, we present plots showing the match between the generated and observed data which allows us to evaluate biases in model fit. Figure S1 presents scatterplots of mean proportion of correct responses, and for the different quantiles of the reaction time distribution (5 to 95 percent quantiles) in the change detection tasks for younger and older adults. Figure S2 shows the data of the delayed estimation tasks. Values closer to the diagonal line indicate a perfect match between the estimated and observed data pattern.

For the change detection tasks, Figure S1 indicate a good model fit for both the reaction time distribution and the average proportion of correct responses. There is a slight tendency that reaction times are underestimated especially for the tail of the reaction time distribution that is captured by the 50 to 95 percent quantiles. Although this kind of a bias in not ideal with respect to model fit, it has been documented before (van Ravenzwaaij & Oberauer, 2009). The simplifications introduced by the EZ-diffusion model versions are known to not fully capture the full reaction time distribution, however they improve precision in parameter estimation, thus increasing the chance to detect empirical effects (van Ravenzwaaij et al., 2017). Van Ravenzwaaij et al. (2017) showed that although the absolute values computed via the EZ-diffusion model may not fully capture the true data, the EZ-diffusion model still adequately captures differences in the experimental effects or between groups. As the present manuscript focused on age comparisons in experimental effects and individual differences, EZ-diffusion parameter will provide an adequate reflection of the effects on the true underlying parameters.

For the delayed estimation tasks, Figure S2 indicated an acceptable model fit. Although rank stability is given, the tendency to underestimate reaction times is stronger in the delayed estimation tasks than for the change detection tasks. Such biases in estimation have been documented for the EZ-circular diffusion model too (Qarehdaghi & Amani Rad, 2022), especially with low trial numbers. An additional reason for this bias might be that participants were not instructed to make a single ballistic movement from the center of the screen to the perimeter when selecting their response. In the original publication of the circular diffusion model (Smith, 2016) this was done in order to separate decision time from non-decision time. Additionally, we were unable to empirically identify guessing trials (e.g., by confidence ratings) that might stem from a different response process compared to trials with proper retrieval of information from memory. Both these issues might have amplified biases in capturing the reaction time distribution. However, as there were no systematic differences between experimental conditions and age groups in this bias, it is unlikely that the bias in model fit affected condition or group differences.

In sum, both the EZ-diffusion and the EZ-circular diffusion acceptably fit the observed data. Yet, given the biases in fitting the response time distributions, especially in delayed estimation tasks, future studies should aim to replicate the results reported in the current study trying to separate decision from non-decision time by carefully instructing response selection in the continuous reproduction tasks.

## Figure S1

### Scatterplots Relating the Observed Data and the Synthetic Data Generated based on the Computed Diffusion Model Parameters for the Change-Detection Tasks. The Diagonal Gray Line Indicates Perfect Fit. Different Colors Mark the Different Materials (Color vs. Orientation), and the Different Shapes the two Experimental Conditions (No-Cue vs. Retro-Cue). MeanPC = Mean Proportion Correct, q05 to q95 = 5 to 95 Percent Quantile of Reaction Times.


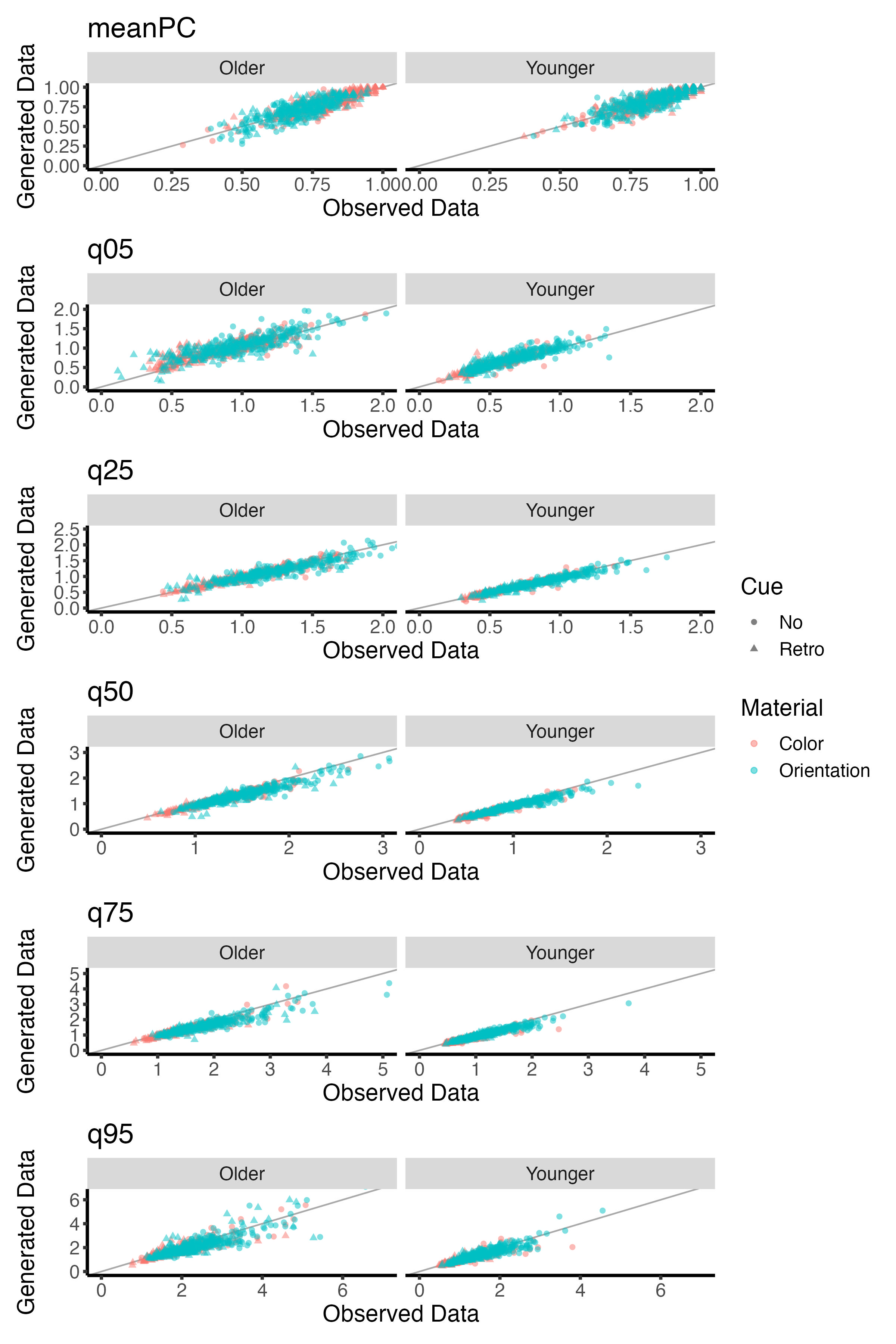


## Figure S2

### Scatterplots Relating the Observed Data and the Synthetic Data Generated based on the Computed Diffusion Model Parameters for the Delayed Estimation Tasks. The Diagonal Gray Line Indicates Perfect Fit. Different Colors Mark the Different Materials (Color vs. Orientation), and the Different Shapes the two Experimental Conditions (No-Cue vs. Retro-Cue). MeanPC = Mean Proportion Correct, q05 to q95 = 5 to 95 Percent Quantile of Reaction Times.


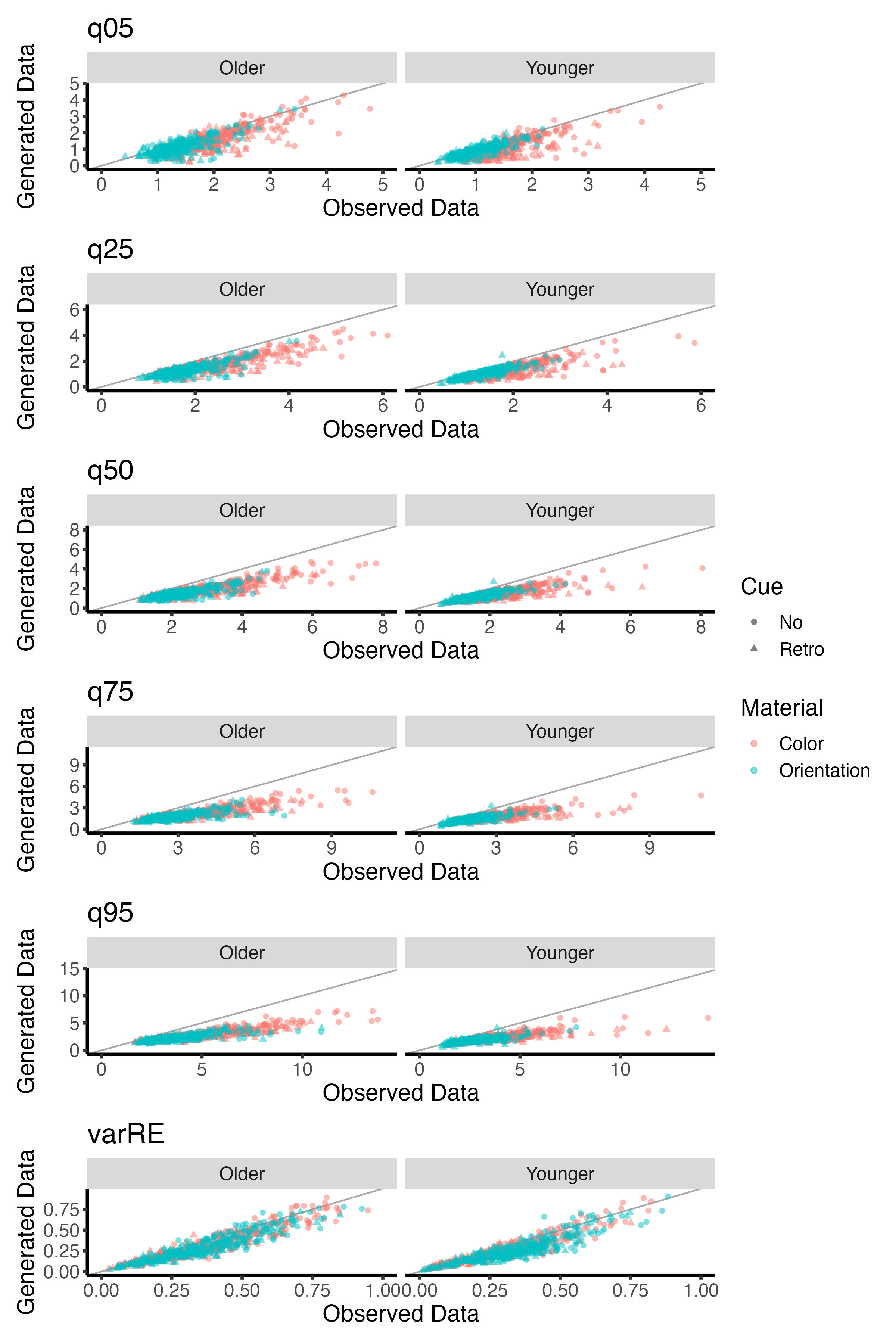


References

Qarehdaghi, H., & Amani Rad, J. (2022). An EZ-circular diffusion model of continuous decision processes. *Proceedings of the Annual Meeting of the Cognitive Science Society*, *44*(44). https://escholarship.org/uc/item/5z09c72m

Smith, P. L. (2016). Diffusion theory of decision making in continuous report. *Psychological Review*, *123*(4), 425–451. https://doi.org/10.1037/rev0000023

van Ravenzwaaij, D., Donkin, C., & Vandekerckhove, J. (2017). The EZ diffusion model provides a powerful test of simple empirical effects. *Psychonomic Bulletin & Review*, *24*(2), 547–556. https://doi.org/10/gd3vsj

van Ravenzwaaij, D., & Oberauer, K. (2009). How to use the diffusion model: Parameter recovery of three methods: EZ, fast-dm, and DMAT. *Journal of Mathematical Psychology*, *53*(6), 463–473. https://doi.org/10/bqh7dp
